# Mitochondrial Dysfunction and Kidney Stone Disease

**DOI:** 10.3389/fphys.2020.566506

**Published:** 2020-10-20

**Authors:** Sakdithep Chaiyarit, Visith Thongboonkerd

**Affiliations:** Medical Proteomics Unit, Office for Research and Development, Faculty of Medicine Siriraj Hospital, Mahidol University, Bangkok, Thailand

**Keywords:** antioxidant, calcium, calcium oxalate, mitochondria, nephrolithiasis, oxidative stress, reactive oxygen species, urolithiasis

## Abstract

Mitochondrion is a pivotal intracellular organelle that plays crucial roles in regulation of energy production, oxidative stress, calcium homeostasis, and apoptosis. Kidney stone disease (nephrolithiasis/urolithiasis), particularly calcium oxalate (CaOx; the most common type), has been shown to be associated with oxidative stress and tissue inflammation/injury. Recent evidence has demonstrated the involvement of mitochondrial dysfunction in CaOx crystal retention and aggregation as well as Randall’s plaque formation, all of which are the essential mechanisms for kidney stone formation. This review highlights the important roles of mitochondria in renal cell functions and provides the data obtained from previous investigations of mitochondria related to kidney stone disease. In addition, mechanisms for the involvement of mitochondrial dysfunction in the pathophysiology of kidney stone disease are summarized. Finally, future perspectives on the novel approach to prevent kidney stone formation by mitochondrial preservation are discussed.

## Introduction

Mitochondrion is a unique and dynamic intracellular organelle that varies in shape, size, and number among various cell types. It is the only subcellular organelle that has double membranes and contains its own genome. Unlike nuclear DNA, mitochondrial DNA (mtDNA) is circular double-stranded DNA that is not protected by histones (Sharma and Sampath, 2019; Yan et al., 2019). Generally, mitochondria are the foremost generator of cellular energy in human cells by converting oxygen and nutritional elements to adenosine triphosphate (ATP) *via* oxidative phosphorylation pathway. Tricarboxylic acid (TCA) cycle (Kreb’s cycle or citric acid cycle) and electron transport chain (ETC) are the two major metabolic processes that play pivotal roles in this energy production mechanism (Brookes et al., 2004; Pieczenik and Neustadt, 2007). Additionally, mitochondria also involve regulation of cellular oxidative stress, calcium homeostasis, apoptosis-signaling pathway, and aging processes in all cells and tissues (Bhargava and Schnellmann, 2017; Duann and Lin, 2017). Mitochondrial dysfunction, therefore, can lead to development of various metabolic diseases and other disorders, such as diabetes (Forbes and Thorburn, 2018; Ducasa et al., 2019), obesity (Song et al., 2017; Schottl et al., 2020), aging (Wojtovich et al., 2012; Giorgi et al., 2018; Jang et al., 2018; Son and Lee, 2019), cancers (Anderson et al., 2018; Neagu et al., 2019; Missiroli et al., 2020), myopathies (Walters et al., 2012; Apostolopoulou et al., 2015), and neurodegenerative disorders (Muller et al., 2018; Rango and Bresolin, 2018; Takahashi and Takahashi, 2019). Interestingly, kidney stone disease (nephrolithiasis or urolithiasis) has been shown to be associated, either directly or indirectly, with mitochondrial dysfunction (Cao et al., 2004; Williams et al., 2016; Patel et al., 2018; Dominguez-Gutierrez et al., 2020).

This review highlights the important roles of mitochondria in renal cell functions and emphasizes associations between mitochondrial dysfunction and the pathophysiology of kidney stone disease as well as the potential for the novel approach to prevent kidney stone formation by preservation of mitochondrial functions.

## Roles of Mitochondria in Renal Cell Functions

The kidney is the second organ after the heart that contains the largest number of mitochondria per cell (Wang et al., 2010; Forbes, 2016). This organ and its related urinary tract system require sufficient energy for body homeostasis, blood filtration, nutrient reabsorption, regulation of body fluid and electrolyte, acid-base balance, and blood pressure regulation (Bhargava and Schnellmann, 2017). The most important source of cellular energy is generated by mitochondria, which are the crucial intracellular organelles to support the renal cell functions. In particular, proximal renal tubular cells are the mitochondria-rich cells that have higher density of mitochondria than other cells lining along the nephron because they require much more energy for their functions, including but not limited to reabsorption of water, glucose, ions, and nutrients (Forbes, 2016). Also, intercalated cells of the collecting ducts are the other mitochondria-rich cells playing roles in acid-base balance and regulation of sodium, chloride, and potassium transports (Roy et al., 2015).

### Energy Production

ATP molecules are generated mainly by electron transfer across the mitochondrial inner membrane (MIM) *via* ETC during oxidative phosphorylation. In addition, TCA cycle also plays pivotal roles in mitochondrial energy production (Forbes, 2016). The TCA cycle produces the following coenzymes in mitochondrial matrix, including three molecules of nicotine adenine dinucleotide (NADH) and one molecule of flavin adenine dinucleotide (FADH_2_), from acetate in the form or acetyl coenzyme A (acetyl CoA), which is converted from pyruvate *via* pyruvate dehydrogenase after glycolysis of one molecule of glucose (Pieczenik and Neustadt, 2007; Ralto et al., 2020). In addition to glycolysis, fatty acid oxidation is another mechanism that can produce even more acetyl CoA molecules for entering into the TCA cycle. As a result, fatty acid oxidation generates more NADH and FADH_2_ molecules compared to glycolysis (Sharpe and McKenzie, 2018). Electrons from NADH and FADH_2_ are transferred to NADH dehydrogenase (complex I) and succinate dehydrogenase (complex II), respectively, of ETC in the MIM. The electrons were then shuttled to ubiquinol-cytochrome c reductase (complex III) by ubiquinone (coenzyme Q10; CoQ10) and transferred to cytochrome c oxidase (COX; complex IV) by cytochrome c (Bhargava and Schnellmann, 2017). During electron transfer through the ETC in the MIM, protons are actively pumped by complexes I, III, and IV through mitochondrial intermembrane space. Finally, ATP synthase (complex V) uses excess protons in mitochondrial intermembrane space to phosphorylate adenosine diphosphate (ADP) to ATP (Bhargava and Schnellmann, 2017). The requirement for ATP in the kidney is cell type-specific as tubular cells need high energy to mediate the active transport function inside the renal cortex. In contrast, cells in the glomerular segment require lower energy for filtration and other passive processes. Overall, a large number of mitochondria and their high activities are required by renal tubular cells to maintain several renal functions.

### Regulation of Oxidative Stress

Oxidative stress is mostly induced by excessive cytoplasmic and mitochondrial reactive oxygen species (ROS) emission (Khand et al., 2002; Duann and Lin, 2017). Electron transfer at complexes I and III has been proposed as the main source of ROS overproduction (Ray et al., 2012). Mitochondria can consume oxygen for ATP production, leading to generation of ROS such as superoxide (O_2_^−^) and hydrogen peroxide (H_2_O_2_). These ROS are important for cellular signaling pathways as well as cell cycles (Deng et al., 2003; Chang et al., 2005; An et al., 2013), apoptosis (An et al., 2013), protein kinases, and protein phosphatase (Brookes et al., 2002). Under physiologic condition, mitochondria have highly efficient antioxidant systems to keep the balance with ROS (Stowe and Camara, 2009). For example, superoxide dismutase rapidly reduces O_2_^−^ to H_2_O_2_ faster than the rate of O_2_^−^ production (Bhargava and Schnellmann, 2017). Subsequently, glutathione peroxidase completes the reduction processes by converting H_2_O_2_ to water (Fernandez-Checa et al., 1998; Ribas et al., 2014). During oxidative stress (pathological condition), excessive ROS emission occurs because the mitochondrial scavenging system is overwhelmed by the perpetual increase in ROS production, the so-called ROS-induced ROS release (RIRR; Zorov et al., 2014). As a result, mitochondrial functions are altered, leading to mtDNA damage and oxidative modifications of mitochondrial proteins and enzymes in TCA cycle and ETC (Khand et al., 2002; Bhargava and Schnellmann, 2017; Duann and Lin, 2017).

In oxidative stress condition, the nuclear factor erythroid 2-related factor 2 (NRF2) can trigger transcription factors of genes encoding antioxidant enzymes to cope with the ROS overproduction (Ruiz et al., 2013). This underscores the important regulatory roles of the mitochondrial antioxidant systems to maintain intracellular ATP production for all cellular events and to preserve mitochondrial functions. In particular, glutathione redox cycle is the critical antioxidant mechanism found in cytoplasm and intracellular organelles, such as nucleus, endoplasmic reticulum, and mitochondria (Ribas et al., 2014; Reiter et al., 2018). Mitochondrial glutathione (mGSH) is the reduced form of glutathione found in mitochondrial matrix and can be oxidized to glutathione disulfide (GSSG) by superoxide anions (Fernandez-Checa et al., 1998; Ribas et al., 2014). GSSG can be reversed to mGSH by glutathione reductase that requires NADPH from the pentose phosphate pathway (Baudouin-Cornu et al., 2012; Lushchak, 2012; Bajic et al., 2019). These two mechanisms are the pivotal processes for preventing oxidative stress and preserving mitochondrial functions.

### Regulation of Apoptosis

Mitochondria have been found to also involve program cell death or apoptosis (Scatena, 2012). The apoptotic cell death is considered when the cell morphology/biology is changed with membrane blebbing, cell shrinkage, and nuclear DNA fragments (Indran et al., 2011). Eventually, apoptotic cells are eliminated by neighboring and/or immune cells to avoid tissue inflammation and damage. Apoptotic cell death mechanism has two main pathways, intrinsic (*via* mitochondria) and extrinsic (*via* death receptors) pathways (Grancara et al., 2016; Abate et al., 2020). However, activation of caspases is the common and key final process that both pathways share together. Caspases are classified into two groups, based on their activities, as the initiator caspases (caspase-2, -8, -9, and -10) and the effector caspases (caspase-3, -6, and -7).

In the intrinsic pathway, mitochondrial cytochrome c is released to the cytoplasm and binds with apoptotic protease activating factor 1 (APAF-1) and ATP, resulting in recruitment of procaspase-9 to form an apoptosome, in which procaspase-9 is cleaved to caspase-9. The caspase-9 then converts procaspase-3 to effector caspase-3, resulting in the completion of cellular apoptosis (Pradelli et al., 2010). For the extrinsic pathway, extracellular ligands bind to the death receptors, leading to formation of death-inducing signaling complex that then cleaves procaspase-8 to caspase-8 (Franklin, 2011). The caspase-8 can directly stimulate the effector caspase-3 that subsequently degrades a variety of proteins during apoptosis. Moreover, the effector caspase-8 has been reported to induce cytochrome c release from mitochondria by increasing permeability of mitochondrial outer membrane (MOM) (Elmore, 2007; Indran et al., 2011). Using both intrinsic and extrinsic pathways, mitochondria thus serve as the important intracellular organelles for regulation of the apoptotic cell death.

## Previous Investigations of Mitochondria in Kidney Stone Disease

Previous studies have shown that the pathogenic processes of kidney stone disease are associated with oxidative stress condition (Hirose et al., 2010; Niimi et al., 2012). The involvement of oxidative stress in kidney stone disease has been found in various *in vitro* studies (Chaiyarit and Thongboonkerd, 2012; Peerapen et al., 2018) and animal models (Hirose et al., 2010; Niimi et al., 2014) as well as stone formers (patients with kidney stones) (Ma et al., 2014; Ceban et al., 2016). Such oxidative induction leads to several downstream cascades, particularly inflammatory response and tissue injury (Williams et al., 2016; Yasui et al., 2017; Dominguez-Gutierrez et al., 2020). More importantly, there is increasing evidence demonstrating that the tissue injury induced by oxidative stress enhances retention of the causative crystals [especially calcium oxalate (CaOx)] inside renal tubules and/or kidney interstitium (parenchyma) that is one of the crucial steps for kidney stone formation (Cao et al., 2004; Khan, 2013, 2014). While a number of reports have shown overproduction of ROS in renal tubular epithelial cells followed by cellular injury, many antioxidant compounds have been introduced to cope with such oxidative stress, to reduce cellular/tissue injury, and to inhibit kidney stone formation (Muthukumar and Selvam, 1998; Itoh et al., 2005; Holoch and Tracy, 2011; Fishman et al., 2013; Zhai et al., 2013; Yang et al., 2016; Zeng et al., 2019). Because cellular oxidation is mostly associated with mitochondrial activities, mitochondria are thus directly involved in such cellular oxidative stress.

Calcium is the most common cation that is precipitated in the urine as the crystalline forms with other anions, especially oxalate and phosphate. Such calcium-containing crystals are commonly found in the urine of kidney stone formers (Yasui et al., 2017). Among all chemical types of kidney stones, CaOx is the most common one found worldwide (Vinaiphat et al., 2017; Vinaiphat and Thongboonkerd, 2017). After crystallization, CaOx crystals can adhere on the surface of renal tubular cells and are then internalized into the cells *via* macropinocytosis (Kanlaya et al., 2013) for subsequent elimination through degradation and/or dissolution (Chaiyarit et al., 2016). The end products of such elimination processes are free calcium and oxalate ions. When the intracellular calcium level is changed, mitochondria play role as one among other mechanisms to regulate intracellular calcium homeostasis. Normally, calcium can promote mitochondrial energy production. On the other hand, calcium overload may cause mitochondrial dysfunction and ROS overproduction (Brookes et al., 2004; Muller et al., 2018). The excess ROS can cause mtDNA damage followed by alterations in mitochondrial fission/fusion process, leading to cellular injury, apoptosis, inflammatory response, and finally the disease progression (Brookes et al., 2004; Suarez-Rivero et al., 2016; Srinivasan et al., 2017; Muller et al., 2018; Yan et al., 2019).

Mitochondrial dysfunction has been recognized as one of the important keys in the etiology of kidney stone disease nearly four decades ago (Laxmanan et al., 1986; Harrison et al., 1988). Calcium dense deposits have been demonstrated inside the enlarged mitochondria in renal tubular cells of the stone formers by electron microscopy without crystalline structure observed (Harrison et al., 1988). Several other investigations have also provided evidence showing the ability of mitochondria to bind with oxalate and CaOx crystals (Laxmanan et al., 1986; Govindaraj and Selvam, 2001, 2002; Selvam and Kalaiselvi, 2003; Hirose et al., 2012; Kohri et al., 2012; Roop-Ngam et al., 2012). The binding mechanism between mitochondria and CaOx crystals has been suggested to be mediated through peroxidation of mitochondrial proteins and lipids, which further promote nucleation and aggregation of CaOx crystals (Govindaraj and Selvam, 2001). The involvement of mitochondria in early phase of kidney stone formation has gained a wide attention because mitochondrial proteins have been found in stone matrices (Govindaraj and Selvam, 2001, 2002) and the mitochondrial fragments have been found together with the crystals along distal renal tubular lumens, suggesting their roles in crystal nucleation (Hirose et al., 2012).

Although mitochondria have been found to directly interact with oxalate ion and CaOx crystals, previous investigations of mitochondria have focused only to their roles in regulation of oxidative stress and tissue injury that commonly occur in kidney stone disease. CaOx crystals have been found to induce oxidative stress in renal tubular cells leading to mitochondrial dysfunction and renal cell injury (Khan, 2013, 2014). Renal tubular cell injury and the defective mitochondria and other intracellular organelles are evidence of aggravated CaOx crystal retention inside the renal tissue, which is considered as one of the important steps for kidney stone development (Govindaraj and Selvam, 2002; Yasui et al., 2017). Following this line of investigations, several previous studies on kidney stone disease thus explored the effects of mitochondrial injury, ROS overproduction, loss of the mitochondrial membrane potential, and mitochondrial swelling (Jonassen et al., 2005; Mcmartin, 2009; Chaiyarit and Thongboonkerd, 2012; Niimi et al., 2012; Yasui et al., 2017; Peng et al., 2019; Wu et al., 2019). Moreover, CaOx crystals can induce production of mitochondrial O_2_^−^ that further activates cyclophilin D (CypD), which is a component of mitochondrial permeability transition pore (mPTP) (Niimi et al., 2012). CypD plays a role in opening such pore and thus affects the permeability of mitochondria at MIM and MOM (Niimi et al., 2012). Additionally, CaOx induces cytochrome c release to cytoplasm for further activation of caspases and related signaling pathways, resulting in apoptosis of renal tubular cells (Jonassen et al., 2003; Niimi et al., 2012). On the other hand, mGSH maintains the mitochondrial integrity and functions, and can also reduce oxalate deposition in hyperoxaluria condition (Muthukumar and Selvam, 1998). *Vice versa*, mGSH reduction can induce mitochondrial dysfunction and may contribute to the development of CaOx kidney stones (Muthukumar and Selvam, 1998).

Our previous mitochondrial proteome study has highlighted the response of renal tubular epithelial cells of the distal nephron segment to CaOx crystals (Chaiyarit and Thongboonkerd, 2012). Using two-dimensional gel electrophoresis followed by quadrupole time-of-flight tandem mass spectrometry (Q-TOF MS/MS), a total of 15 mitochondrial proteins were identified to have differential expression levels after the distal renal tubular cells were exposed to CaOx crystals. Among these, proteins that played roles in maintaining mitochondrial functions (i.e., pyruvate dehydrogenase, ATP synthase, and NADH dehydrogenase) and cell death (ezrin) were increased (Chaiyarit and Thongboonkerd, 2012). In combination with comprehensive bioinformatics analysis of other large proteome datasets together with functional validation, the additional results indicate the association between mitochondrial dysfunction and oxidative stress-induced renal tubular cell injury (Peerapen et al., 2018).

Mitochondria have also been investigated in Randall’s plaque model of kidney stone pathogenesis. Mitochondrial dysfunction has been found to be related not only to renal tubular injury but also to the impaired immune response and inflammation by decreasing monocytes’ mitochondrial functions in the CaOx stone formers, leading to the decline of crystal elimination that further enhances tissue inflammation (Williams et al., 2016; Patel et al., 2018; Dominguez-Gutierrez et al., 2020). Progressive tissue inflammation together with supersaturation of calcium phosphate then induces Randall’s plaque formation and finally kidney stone development (Williams et al., 2016; Patel et al., 2018; Dominguez-Gutierrez et al., 2020). To cope with the impaired immune response and tissue inflammation as well as the interstitial plaque development, various antioxidants or free radical scavengers (Muthukumar and Selvam, 1998; Kohri et al., 2012; Zhai et al., 2013; Aggarwal et al., 2016; Chhiber et al., 2016) and polysaccharide compounds (Veena et al., 2007; Sun et al., 2016; Guo et al., 2018; Sun et al., 2019) have been shown to serve as the therapeutic/preventive strategies to rescue/prevent kidney stone formation.

## Mechanisms for the Involvement of Mitochondrial Dysfunction in Pathophysiology of Kidney Stone Disease

Oxalate and calcium can alter mitochondrial activities, leading to changes in metabolic status that may induce loss or alterations of mitochondrial functions on energy production, ROS regulation, and intracellular calcium homeostasis, all of which affect mitochondrial biogenesis (Veena et al., 2008; Hirose et al., 2010; Niimi et al., 2012; Sun et al., 2017). The dynamic processes between mitochondrial fusion and fission generate various by-products, most of which are mitochondrial fragments and ROS (Aparicio-Trejo et al., 2018; Janikiewicz et al., 2018; Geto et al., 2020). As aforementioned, several lines of evidence have suggested the involvement of mitochondrial dysfunction in the initial phase of kidney stone disease. There are three main mechanisms proposed for the involvement of mitochondrial dysfunction in the pathophysiology of kidney stone disease.

### Mechanism I

**Figure 1:**
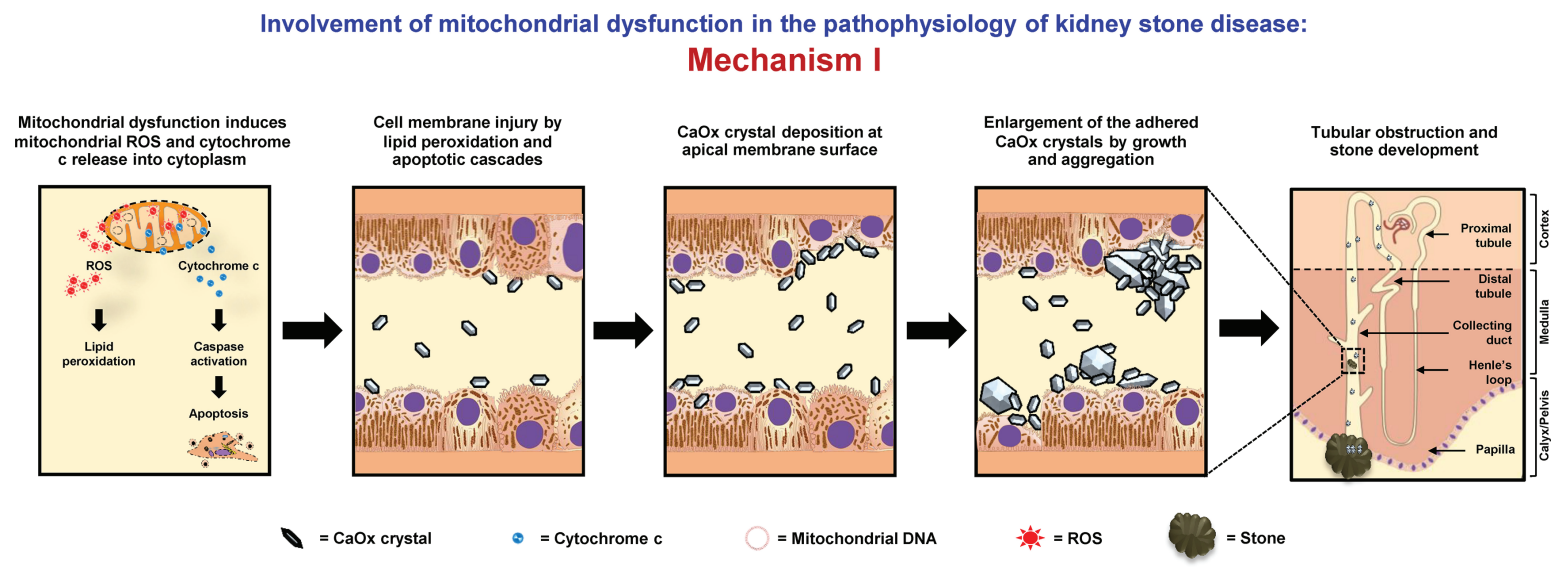
Involvement of mitochondrial dysfunction in the pathophysiology of kidney stone disease: *Mechanism I* – Mitochondrial dysfunction induces the release of mitochondrial reactive oxygen species (ROS) and cytochrome c to cytoplasm. The lipid peroxidation and apoptotic cascades can then cause cell membrane injury that enhances calcium oxalate (CaOx) crystal deposition on apical surfaces of renal tubular cells of the distal nephron segment. The accumulated crystals can be further enlarged and self-aggregated, leading to the stone formation. The right panel shows the macroscopic diagram to demonstrate the locales (related to the nephron segments) where the proposed mechanism occurs.

Mitochondrial dysfunction can increase retention of CaOx crystals inside renal tubular lumens of the distal nephron segment. These stagnant crystals subsequently form the stone nidus, which is the central part of the stone generated from crystal aggregates that finally become the macroscopic stone. The loss or defect of mitochondrial energy production can enhance metabolic process in TCA cycle and ETC, resulting in the increase of ROS production and reduction of antioxidant enzymes (Brookes et al., 2004; Chaiyarit and Thongboonkerd, 2012; Peerapen et al., 2018). Consequently, the excess mitochondrial free radicals can damage mitochondrial membranes (MIM and MOM). As a result, mitochondrial ROS, cytochrome c, calcium, and other proinflammatory factors are further released to the cytoplasm (Cao et al., 2016; Fong-Ngern et al., 2017). High level of cytoplasmic ROS can induce lipid peroxidation, which damages cell membranes and further enhances crystal deposition on apical surfaces of renal tubular cells (Cao et al., 2016; Fong-Ngern et al., 2017). Additionally, apoptotic signaling cascades are activated by cytochrome c that also upsurges renal tubular cell injury, leading to crystal adhesion and intrarenal crystal retention (Cao et al., 2016; Fong-Ngern et al., 2017). The accumulated crystals can be further enlarged and self-aggregated, leading to the stone formation (Figure 1).

### Mechanism II

**Figure 2:**
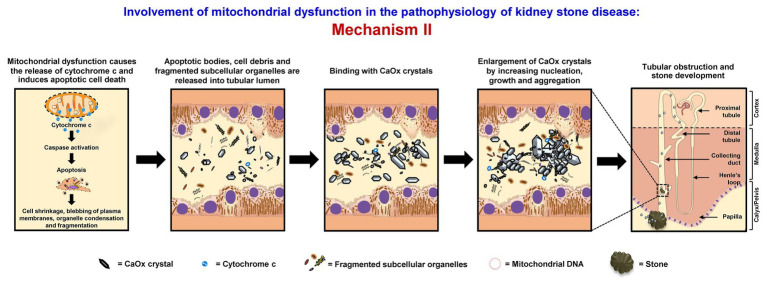
Involvement of mitochondrial dysfunction in the pathophysiology of kidney stone disease: *Mechanism II* – Mitochondrial dysfunction causes the release of cytochrome c and induces apoptotic cell death associated with cell shrinkage, blebbing of plasma membranes, organelle condensation, and fragmentation. The resulting apoptotic bodies, cell debris, and fragmented subcellular organelles are released into tubular lumens and can then bind with CaOx crystals. Moreover, fragmented mitochondria and mitochondrial proteins together with the membrane fragments and other cellular debris can serve as the raw materials for the stone nidus formation in the distal nephron and further promote crystal nucleation, growth, and aggregation. The large aggregates may obstruct tubular lumen and/or migrate (by renal tubular fluid flow) to the calyx and pelvis to form the stone. The right panel shows the macroscopic diagram to demonstrate the locales (related to the nephron segments) where the proposed mechanism occurs.

Mitochondrial dysfunction causes the release of cytochrome c and induces apoptotic cell death, associated with cell shrinkage, blebbing of plasma membranes, organelle condensation, and fragmentation. Thereafter, apoptotic bodies, cell debris, and fragmented subcellular organelles are released into tubular lumen and then bind with oxalate and CaOx crystals as shown by several studies (Govindaraj and Selvam, 2001, 2002; Selvam and Kalaiselvi, 2003; Hirose et al., 2012; Kohri et al., 2012). Also, fragmented mitochondria and mitochondrial proteins (i.e., 48-kDa protein) have been found in the stone core matrices and thus may get involved in the stone nidus formation (Govindaraj and Selvam, 2001, 2002). These mitochondrial components, membrane fragments, and other cellular debris can directly serve as the raw materials for the stone nidus formation in the distal nephron segment and further promote crystal nucleation, growth, and aggregation. The large aggregates may obstruct tubular lumen and/or migrate (by renal tubular fluid flow) to the calyx and pelvis to form the stone (Evan, 2010; Khan et al., 2016) (Figure 2).

### Mechanism III

**Figure 3:**
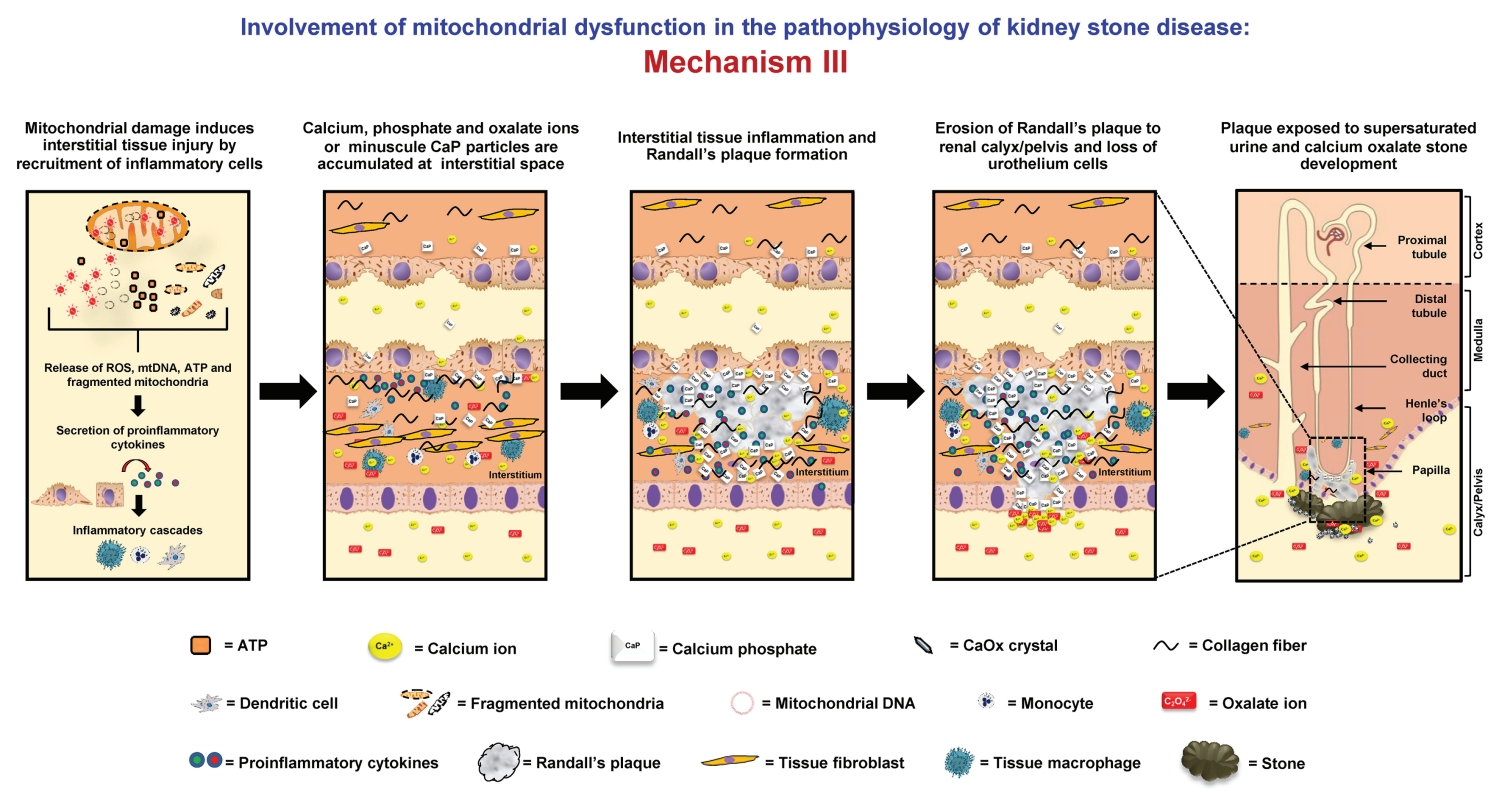
Involvement of mitochondrial dysfunction in the pathophysiology of kidney stone disease: *Mechanism III* – Mitochondrial damage induces the disruption of mitochondrial membrane integrity followed by the release of ROS, mitochondrial DNA (mtDNA), adenosine triphosphate (ATP), and fragmented mitochondria to the cytoplasm. These components can then induce secretion of the proinflammatory cytokines, trigger the inflammatory cascades, and further enhance interstitial tissue inflammation. Together with supersaturation of calcium phosphate, which is common in the renal interstitium, Randall’s plaque containing mainly calcium phosphate (hydroxyapatite) starts to form. Some of these plaques can erode into the urinary space or renal pelvis, where supersaturation of CaOx is very common in the stone formers. At this locale (mostly lateral to the renal papilla), the Randall’s plaque can serve as the nidus or stem for further development or growth of CaOx stone. The right panel shows the macroscopic diagram to demonstrate the locales (related to the nephron segments) where the proposed mechanism occurs.

Mitochondrial dysfunction can initiate interstitial stone formation (Randall’s plaque model). In oxidative stress condition, mitochondrial damage can lead to disruption of mitochondrial membrane integrity followed by the release of ROS, mtDNA, ATP, and fragmented mitochondria to the cytoplasm. These components can then induce secretion of the proinflammatory cytokines and trigger the inflammatory cascades (West, 2017). The proinflammatory cytokines that are secreted in response to mitochondrial damage also recruit various immune cells, including tissue macrophages, to this interstitial locale. Migration of these inflammatory cells can cause tissue inflammation (Singhto et al., 2013, 2018). Moreover, the renal interstitium is commonly supersaturated with calcium phosphate, which is another common crystalline compound found in kidney stones. Together with tissue inflammation, Randall’s plaque rich with calcium phosphate starts to form (Khan, 2013, 2014). Some of these plaques can erode into the urinary space or renal pelvis, where supersaturation of CaOx is very common in the stone formers. At this locale (mostly lateral to the renal papilla), Randall’s plaque can serve as the nidus or stem for further development or growth of CaOx stone (Khan et al., 2016; Bird and Khan, 2017; Wiener et al., 2018) (Figure 3).

## Conclusions and Perspectives

In summary, mitochondria may be considered as the central intracellular organelles that play pivotal roles in kidney stone pathophysiology. Alterations in their main functions, including energy production and regulation of oxidative stress and intracellular calcium homeostasis, are associated with kidney tissue injury and inflammatory response, leading to CaOx crystal nucleation, growth, aggregation, and deposition that are the key processes for kidney stone formation. Furthermore, mitochondrial fragmented products and proteins can bind directly with CaOx crystals and thus play roles in the stone nidus formation. In addition to the intratubular crystal deposition and nidus formation, mitochondrial dysfunction is also associated with Randall’s plaque formation by enhancing tissue inflammation and interstitial deposition of calcium phosphate (hydroxyapatite).

It should be noted that mitochondrial dysfunction alone is not sufficient to induce kidney stone formation, of which mechanisms are multifactorial. For example, mtDNA mutations or defective nuclear-encoded mitochondrial proteins have been extensively studied in several mitochondrial disorders, e.g., Alzheimer’s disease, epilepsy, mitochondrial myopathy, encephalopathy, lactic acidosis and stroke-like episodes (MELAS syndrome), etc. (El-Hattab et al., 2015; Annesley and Fisher, 2019). Nevertheless, there is no evidence that link between these mitochondrial disorders and kidney stone disease. Although, MELAS syndrome also affects acid-base balanced regulated by kidney cells, the main target of this mitochondrial disorder is the neurological system, whereas kidney stone formation requires the intrarenal microenvironment involving several factors, e.g., supersaturation of CaOx, calcium phosphate, and other causative crystalline compounds, low urinary flow, imbalance of stone inhibitors and promoters, tubular cells injury, oxidative stress, inflammatory cascades, etc. (Bird and Khan, 2017).

Based on the aforementioned pathogenic mechanisms, recovering the mitochondrial functions by antioxidants may be the effective approach for prevention of new or recurrent kidney stone formation. Although antioxidant agents have been recommended to preserve mitochondrial activities and to antagonize ROS overproduction in various diseases, their efficacy and adverse events are still ambiguous and need to be further elucidated. Additionally, previous evidence has demonstrated that CypD (Duann and Lin, 2017) and Bcl-2 interacting protein 3 (BNIP3) (Chaanine et al., 2016; Peng et al., 2019) are related to mitochondrial dysfunction *via* alterations of mitochondrial membrane activities and death signaling release, respectively. To recover the mitochondrial functions and to reduce oxidative stress, inhibition of CypD activation using cyclosporin A (Duann and Lin, 2017) and N-methyl-4-isoleucine cyclosporine (Niimi et al., 2014) has been reported. In addition, expression and translocation of BNIP3 to mitochondria have been reported as the cell death regulatory factors for mitochondrial dysfunction (Zhang and Ney, 2009). This protein also involves mitochondrial membrane potential, mitochondrial transition pore forming, oxidative stress, calcium homeostasis, and inflammation in various cell types (Gao et al., 2020). Therefore, therapeutic application should be considered to combine antioxidants with other promising compounds (i.e., to inhibit activation of CypD, BNIP3 and other related molecules) for prevention of new and/or recurrence stone formation in the future. More importantly, their efficacies and adverse events must be evaluated in large cohorts.

Although the roles for mitochondrial dysfunction related to oxidative stress and CaOx crystal deposition have been well documented, the roles for excessive calcium that is also common in the stone formers (Canales et al., 2010; Chutipongtanate et al., 2012) may be overlooked. Recently, calcium is recognized as a mitochondrial regulator involving several steps of energy production (Gincel et al., 2001; Calderon-Cortes et al., 2008; Pivovarova and Andrews, 2010; Kaufman and Malhotra, 2014; Ummarino, 2017; Ham et al., 2019; Lambert et al., 2020). On the other hand, mitochondria also play roles in regulation of calcium homeostasis (Gincel et al., 2001; Calderon-Cortes et al., 2008; Pivovarova and Andrews, 2010; Kaufman and Malhotra, 2014; Ummarino, 2017; Ham et al., 2019; Lambert et al., 2020). A previous study on ethylene glycol induced kidney stone disease in rats has shown that only CaOx crystals, but not oxalate ion alone, could weaken mitochondrial functions (Mcmartin and Wallace, 2005). Nevertheless, the association among mitochondrial dysfunction, intracellular or mitochondrial calcium concentration, and the stone pathogenesis remains unclear and should be further elucidated. Having done so, the findings to be obtained may lead to the new strategy to cope with mitochondrial dysfunction during the stone development and ultimately to efficient prevention of kidney stone formation.

## Author Contributions

SC and VT drafted the manuscript, read and approved the final manuscript, and are responsible for all aspects of the manuscript. All authors contributed to the article and approved the submitted version.

### Conflict of Interest

The authors declare that the research was conducted in the absence of any commercial or financial relationships that could be construed as a potential conflict of interest.

## Abbreviations

Acetyl CoA: acetyl coenzyme A
ADP: adenosine diphosphate
ATP: adenosine triphosphate
BNIP3: Bcl-2 interacting protein 3
CaOx: calcium oxalate
CypD: cyclophilin D
ETC: electron transport chain
FADH_2_: flavin adenine dinucleotide
GSSG: glutathione disulfide
H_2_O_2_: hydrogen peroxide
mGSH: mitochondrial glutathione
MIM: mitochondrial inner membrane
MOM: mitochondrial outer membrane
mtDNA: mitochondrial DNA
NADH: nicotine adenine dinucleotide
O_2_^−^: superoxide
ROS: reactive oxygen species
TCA: tricarboxylic acid
